# Rhinosinusitis in a patient with Behçet's syndrome

**DOI:** 10.1016/S1808-8694(15)31320-3

**Published:** 2015-10-20

**Authors:** Lauro João Lobo Alcântara, Alcides Trentin Júnior, Marcos Mocellin, João Gilberto S. Mira, Fábio Robert, Ordival Augusto Rosa

**Affiliations:** 1 Physician, Discipline of Otorhinolaryngology, Hospital de Clínicas- University of Parana -UFPR; 2 Faculty member, University of Parana -UFPR; 3 Head of the Service of Otorhinolaryngology, Hospital de Clínicas- University of Parana -UFPR; 4 Physician, Discipline of Otorhinolaryngology, Hospital de Clínicas- University of Parana -UFPR; 5 Internist, Service of Otorhinolaryngology, Hospital de Clínicas- University of Parana -UFPR; 6 Internist, Service of Otorhinolaryngology, Hospital de Clínicas- University of Parana -UFPR

**Keywords:** rhinossinusitis, vasculitis and Behçet

## Abstract

The condition known as Behçet's syndrome was first described by H. Behçet, a dermatologist from Turkey. Its major component is recurrent aphthous-like lesions of the oral mucosa. Some groups of people such as the Japanese are more prone to develop the condition. Behçet's syndrome is relatively rare in the American continent. In addition to oral lesions, these patients may develop recurrent genital ulcerations, uveitis, and pustular vasculitis of the skin, synovitis, and meningoencephalitis. The diagnosis is based on occurrence of internationally proposed major or/and minor criteria and on their combinations. Treatment is challenging and must be tailored to each patient according to the pattern of organ involvement, often requiring use of combined therapies. The clinical picture of the patients in this study confirmed Behçet's Syndrome diagnosis. Vasculitis was evidenced by the absence of bleeding during the handling of some very bloody potential areas. In the present case, not only the triggering but also the complications of rhinosinusitis (periorbitary abscess) were attributed to Behçet's vasculitis, specially the effects on bloody perfusion and draining. Rhinosinusitis is a potential symptom of Behçet's Syndrome. Physicians must pay attention to it in order to achieve satisfactory outcomes.

## INTRODUCTION

Behçet's syndrome is an inflammatory disorder of unknown etiology that affects blood vessels, primarily venules, and is characterized by recurrent oral and genital ulcer lesions, uveitis, aphtous-like skin lesions and acne like eruptions^1^, ^2^.

There is no isolated infectious agent. Although its etiology is sought as viral there is strong correlation between HLA B51 markers and this syndrome. Thus, the individual that has such marker has 3.8 more chances of developing the disease. Additionally, complement serum levels are generally higher specially those of C9^3^, ^4^.

Behçet's syndrome may occur in several ways, but recurrent aphtous-like oral ulceration symptom is present in 99% of the patients, whereas CNS is involved in approximately 10% of the cases, being a severity indicator^5^, ^6^.

International standardization of diagnosis has been proposed and complete Behçet Syndrome is associated with four major criteria (recurrent aphtous-like oral lesions, eye lesions, genital ulceration and skin lesions). Some minor criteria, however, (arthritis, gastrointestinal lesions, vascular lesions and CNS involvement) are considered. Diagnosis also should be suspected if two major sites are affected^7^, ^8^.

Treatment is complex and requires combined therapy according to the organs involved, some therapies include corticosteroids, immunesuppressing drugs, and chlorambucil, colchicines, sulphasalazine and fibrinolytic agents^7^, ^9^.

Since Behçet's Syndrome is rare, data about it are not significantly supported by literature both quantitatively and qualitatively in order to define, characterize and mainly relate it to Otorhinolaryngology^5^.

Martins et al.^10^ reported the first case in 2003 with association between significant chance and development of rhinitis with destructive sinus disease with Behçet's syndrome.

## CASE REPORT

The patient NMS, female, 11 years old, with former diagnosis of Behçet's Syndrome was admitted to ICU of Hospital Pequeno Príncipe due to convulsive condition. During patient's stay in the hospital disease evolved to facial pain and bilateral purulent rhinorrhea. Since the patient already had severe nasal obstruction, clinical symptoms worsened in four days with fever episodes (38.8º C), and ceftriaxone 30 mg/kg/day was prescribed.

Physical examination was as follows: patient weighted 28kg and was 1 meter tall, had genital ulceration and bleeding mucosa.

Otorhinolaryngological examination showed aphtous-like oral lesions, oropharynx and lower turbine hyperemia, excessive secretion and fibrin in the nasal cavity. The initial protocol was to increase the dosage of ceftriaxone to 60 mg/kg/day.

**Figure 1:**
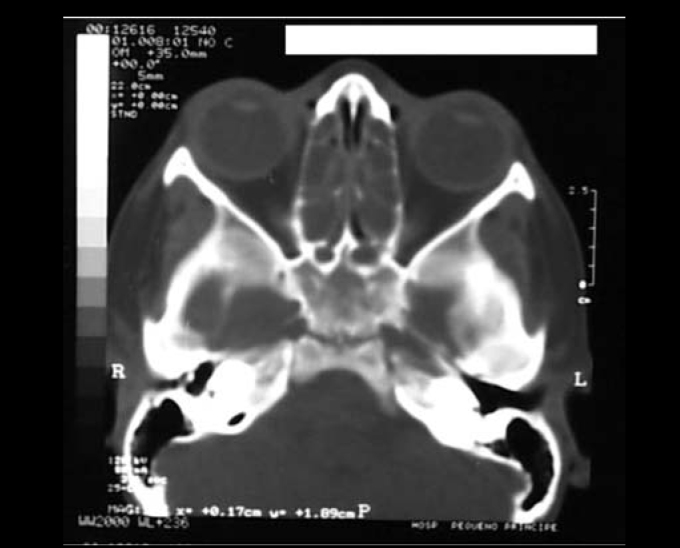
Axial cross-section showing velamentum of maxillary sinuses and ethmoidal cells and bilateral obliteration of infundibulum.

**Figure 2:**
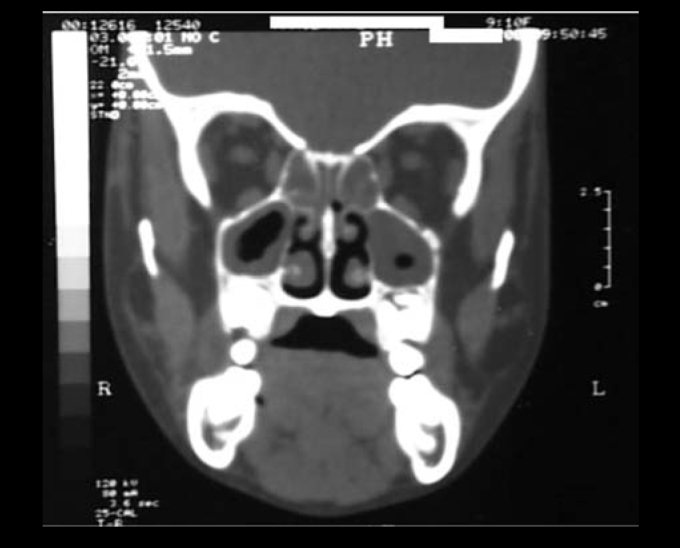
Coronal cross-section showing velamentum of maxillary sinuses and ethmoidal cells and bilateral obliteration of the infundibulum.

After two days, patient evolved to worsening of symptoms with significant headache. Then, CT scan of the facial sinuses was requested (Figures 1 and 2). Ceftriaxone therapy was maintained and patient was reevaluated the next day. A new CT scan was requested and right-sided eyelid edema was detected and patient still had the same complaints. Another CT scan was requested and the images confirmed the existence of periorbitary abscess. Drainage was performed on the same day with incision of the superior medial right orbit and dissection extended to the abscess with a counter incision of the nasal fossa through the ethmoid and placement of a Pen Rose drain. Next, nasal cavity was cleaned to remove any lumps and residues of fibrin, bleeding was almost negligible despite massive lower turbine necrosis (highly bleeding region) suggesting Behçet-related vasculitis.

Patient evolved properly until the second postoperative day with mild improvement of clinical symptoms. In the 3rd day, however, she had and exacerbation of eyelid edema followed by fever (39º Celsius). CT scan of paranasal sinuses was then requested, and the report reinforced the idea that a more comprehensive resection was required. Biopsy of the mucosa of the right maxillary sinus showed: “Necrotic tissue in granulation tissue areas with chronic and unspecific inflammation and massive fibrin deposition”.

Caldwell-Luc surgery was then performed using the maxilla ethmoidectomy technique. The incision was made on the gingival labial mucosa in other to be able to open the anterior wall of the maxillary sinus. Abundant purulent secretion was found to the left and mucosa presented mild thickness. The right side had mucosa secretion and degeneration requiring cleaning since it filled the entire sinus. Additionally partial necrosis of the lower and middle turbines was also found. Ethmoid curettage and maxillary sinus drainage were performed with bilateral nasal counter opening.

The patient had good evolution after that until discharge from hospital. The sequel was bilateral synechia resulting from both the clinical picture and intervention.

## DISCUSSION

The confirmation of Behçet's Syndrome diagnosis was achieved after the patient presented three major criteria (recurrent aphtous-like oral lesions, genital ulceration and eye lesions) and two minor criteria (vascular lesions and CNS involvement – likely etiology of convulsions) of Behçet's Syndrome as internationally standardized criteria.

Martins et al.^10^ published in an unheard-of fashion a likely association between Behçet's Syndrome and rhinosinusopathy in a male patient (47 years old) that also developed chronic bilateral middle ear otitis and CT scan findings were similar to those of the patient of this study (predominantly maxilla-ethmoidal with destructive characteristics). They concluded, however, due to the inexistence of a reliable histological marker, that clear documentation of such association should be postponed.

Other authors^1^, ^11^ found the involvement of the inner ear as a late complication of Behçet's Syndrome (approximately one decade after the onset of the condition), vasculitis (primary pathological lesion of this disease) was considered the only responsible factor for labyrinth disorder. Detailed otology and vestibular examination revealed cochlea and vestibular apparatus abnormalities, in addition to results indicating that 62% of Behçet's Syndrome patients complain of auditory disorders and 37% of them present vertigo symptoms.

Vasculitis as reported by the two authors is a likely explanation of inexpressive bleeding after removing lumps and even fibrin plates while cleaning nasal cavity of the patient in this case. It suggests vascular abnormality such as the key aspect of the pathophysiology of rhinosinusitis reported in this case study.

The vasculitis found in Behçet's Syndrome is characterized by necrotizing inflammatory response of the micro vessels (preferably venules which have build up fragmented neutrophils and lymphocytes) and due to the fact that all lesions are in the same evolution stage and were classified in the group of “Immune Response Vasculitis”^10^.

Poor perfusion resulting from spasmodic contraction of arterial vessel certainly is one of the major consequences of vasculitis that also causes deficient venous drainage^2^. A poorly perfused tissue is more likely to present infection and subsequent inflammation than healthy tissue (mainly nasal and paranasal sinuses). Tissue is also poorly drained since there is a build up of most of the elements and toxic substances resulting from inflammatory reaction of the lesion site itself^2^. Partial necrosis of lower and middle turbines was also mentioned and included several portions of the maxillary sinuses and of ethmoidal cells.

In addition to predisposition to an infectious process, clear complexity of clinical care of vasculitis may arise since it worsens inflammatory symptoms (periorbitary edema), exacerbates disease symptoms (partial necrosis of the turbines) and not only makes it more difficult (patient is refractory to clinical treatment) but also extends the treatment (in this case, 40 days).

In spite of clinical and pathophysiological evidences, the association between vasculitis in Behçet's Syndrome and rhinosinusitis could not be precisely established due to lack of specific histological markers that may be analyzed during biopsy of facial sinuses.

Finally, both the age of the patient and specificity of the symptoms found, particularly the development of Behçet's Syndrome with rhinosinusopathy in the absence of ear disease provided a relative uniqueness to this case report.

## FINAL REMARKS

The patient with Behçet's syndrome is a vulnerable target of innumerable affections that may affect practically all body systems. Certainly, the pathophysiology of this disease still needs to be studied in depth to clarify its forms of action. It is expected, however, that after this paper physicians will pay more attention to the fact that rhinosinusitis could be one of the severe symptoms of such syndrome.
